# Association between asthma and invasive pneumococcal disease risk: a systematic review and meta-analysis

**DOI:** 10.1186/s13223-020-00492-4

**Published:** 2020-11-10

**Authors:** Lingling Li, Yusheng Cheng, Xiongwen Tu, Jie Yang, Chenghui Wang, Min Zhang, Zhiwei Lu

**Affiliations:** 1 Department of Respiratory Medicine, Yijishan Hospital, Wannan Medical College, Wuhu, China; 2 Department of Emergency, Yijishan Hospital, Wannan Medical College, Wuhu, China

**Keywords:** Asthma, Invasive pneumococcal disease, Meta-analysis

## Abstract

**Purpose:**

Asthma has been shown to be related to an increased risk of invasive pneumococcal disease (IPD), although the results remain inconclusive. Therefore, we performed a meta-analysis to determine whether asthma increases the risk of IPD. This meta-analysis was performed to validate and strengthen the association between asthma and IPD.

**Methods:**

PubMed, EMBASE, Web of Science, and the reference lists of all relevant articles and books were screened until May 2019. Two authors independently assessed eligibility and study quality and extracted data. A common odds ratio was estimated using a random-effects meta-analysis model of aggregated published data.

**Results:**

A total of eight studies with 8877 IPD cases and 78,366 controls were included. Our meta-analysis showed that asthma was significantly associated with the increased risk of IPD (OR 2.44 [95% CI, 2.02–2.96]). The children with asthma (0–17 years old) (OR 2.86 [95% CI 1.80–4.55]) had a higher risk of IPD susceptibility compared with the adult patients (≥ 18 years old) (OR 2.45 [95% CI 1.98–3.03]).

**Conclusions:**

Results of this meta-analysis indicated that the patients with asthma had a higher risk of IPD susceptibility, especially among the children with asthma.

## Background

*Streptococcus pneumoniae* is commonly found in the nasopharynx, which causes a broad spectrum of diseases from otitis media and sinusitis, to nonbacteremic pneumonia and, finally, to invasive pneumococcal disease (IPD). Invasive pneumococcal disease includes meningitis, sepsis, and complicated pneumonia, and is a major cause of morbidity and mortality worldwide, particularly among patients with certain undelying illnesses [1]. The risk of invasive pneumococcal disease, infections in which pneumococcus can be isolated from a normally sterile body fluid, is higher for young children and the elderly [2, 3]. Not surprisingly then, the economic burden of pneumococcal disease in the world has been reported to be substantial. Therefore, identification of risk factors in the occurrence and development of IPD is important.

Asthma is a chronic inflammatory disease characterized by airway hyperresponsiveness, airway inflammation, reversible airflow obstruction, and airway wall remodeling [4]. Asthma affects almost 300 million people worldwide, and the prevalence of asthma has increased over the past two decades in both children and adults [5, 6]. Specific chronic conditions reported to be associated with an increased IPD risk include anatomic or functional asplenia, congenital and acquired immunodeficiency, anatomic abnormalities, asthma, and nephrotic syndrome [7–13].

There have been a few studies that have included asthma status in assessing the risk factors for IPD [12, 14–18]. Nevertheless, the impact of asthma on the onset of IPD is not as clearly established and international recommendations do not suggest pneumococcal vaccination in asthmatic patients [19]. Until recently, and to what extent asthmatic patients contribute to the burden of IPD at a population level had not been systematically studied. Hence, estimation of the association between asthma and IPD is necessary. Therefore, we conducted a systematic review and a meta-analysis to clarify and quantify the relation between asthma and IPD, and to provide support for a vaccination policy to prevent IPD for asthmatics in the world.

## Methods

### Data sources and search strategy

A computerized literature search was conducted in MEDLINE, EMBASE, and the Web of Science from their inception to May 1, 2019 by two independent investigators (L.L. and Y.S.C.). The following keywords were used for asthma: asthma, bronchial spasm, bronchoconstriction, bronchial hyperreactivity, airway inflammation, wheeze, and wheezing. The following keywords were used for IPD: pneumococcal infections and invasive pneumococcal disease. To minimize any potential bias, there were no limits on language, population, sample size, or date of publication. We also obtained additional studies from the reference lists of relevant reviews and original articles.

### Selection criteria

In our meta-analysis, the included articles had to meet the following criteria: (1) cohort, case–control, or cross-sectional design; (2) original studies that had an independent study population; (3) studies that provided an adequate definition of IPD; (4) studies that provided an adequate definition of asthma; (5) studies that provided relevant and applicable quantitative information on the relation between asthma and IPD. The exclusion criteria of studies were the following: (1) basic publications, cell or animal models; (2) insufficient data; (3) duplicated report; (4) reviews, comments, abstracts, case report.

### Data extraction

Based on the search strategy and selection criteria, two investigators (L.L. and Y.S.C.) reviewed the titles, abstracts, and full articles to obtain the eligible studies. For each included study, the following data were collected: first author, year, and study population (number, age group, gender, and setting), study design, methods of *Streptococcus pneumoniae* detection and diagnosis, types of specimen, and sample size. The numbers of asthmatics and non-asthmatics in the IPD group and control group of each study were recorded. Disagreement was resolved by discussion.

### Statistical analysis

Odds ratio (ORs) with 95% confidence intervals (CIs) are reported for IPD cases with asthma compared with control subjects. Subgroup analyses were performed by group age and study design to identify the association. The heterogeneity of the studies included in this meta-analysis was assessed using the Q statistic test and the I^2^ statistic test [20]. The random-effects model was selected when* P* value < 0.1 or I^2^ > 50%; otherwise, the fixed-effects model was selected. Possible publication bias was evaluated by visual inspection of funnel plots and application of the Begg test [21]. All statistical analyses were performed using STATA 10.0 (StataCorp, College Station, Texas). A P value less than 0.05 was identified as statistically significant.

## Results

### Literature search

**Fig. 1:**
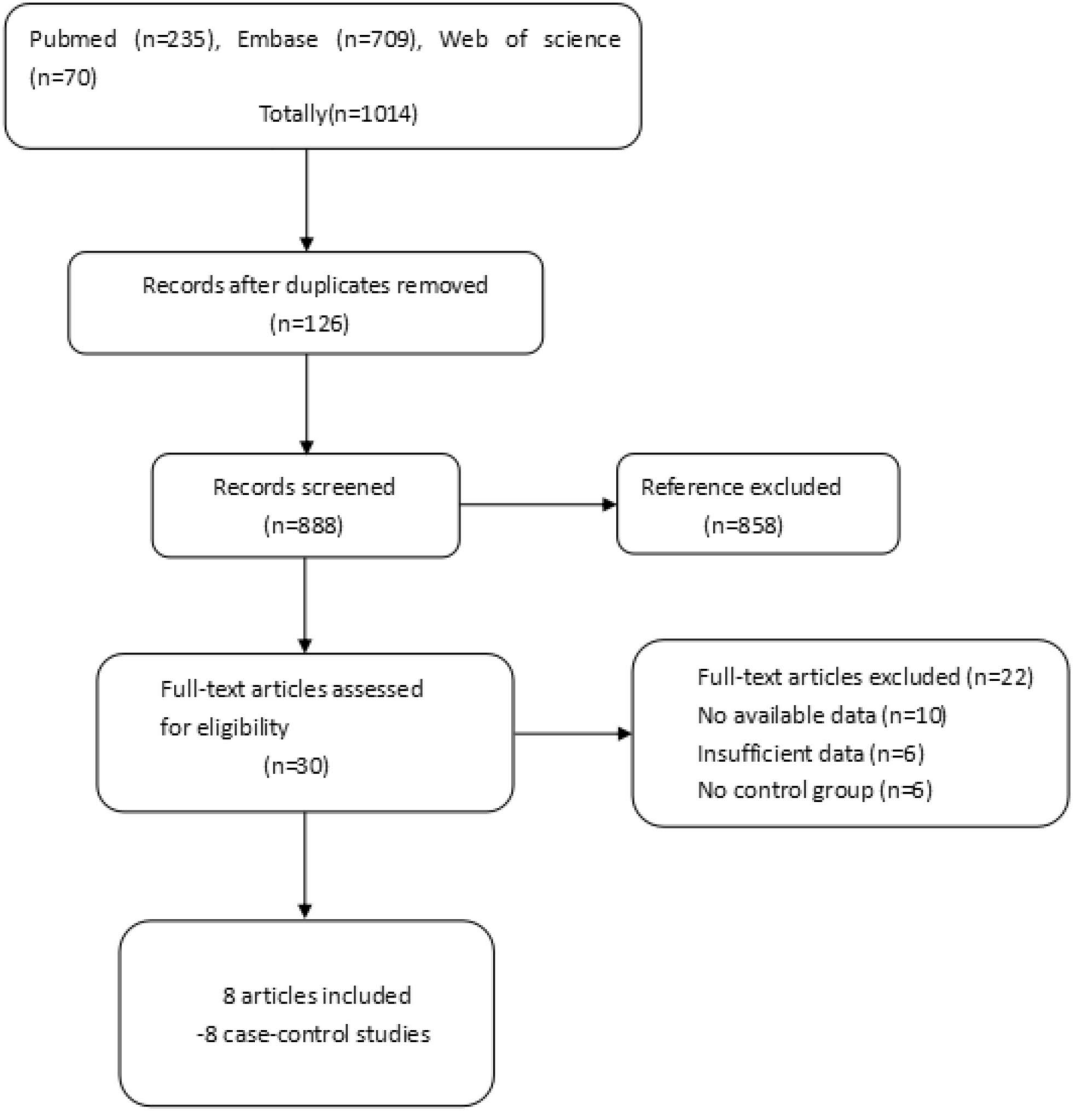
References searched and selection of studies in the meta-analysis

As shown in Fig. 1, a total of 1014 articles were reviewed, of which 858 were irrelevant and 126 were duplicate studies. Thus, these articles were excluded from the study after screening the titles. In the remaining 30 articles, 10 were excluded for lack of available data, 6 were excluded for lack of sufficient data, and 6 were excluded for lack of a control group. Therefore, 8 articles were included in this study, which consisted of 8 case–control studies.

### Characteristics of included studies

Eight studies were included [12, 14, 16, 17, 22–25], providing a total 8877 IPD cases and 78,366 controls to investigate the risk of susceptibility IPD in patients with asthma. In all studies, a case of IPD was defined as an illness in which *S. pneumoniae* was isolated from a normally sterile body fluid (blood, CSF or pleural fluid). Four studies investigated the association between asthma and IPD in adults, three studies provided detailed incidences of asthma in children, and the remain study included patients of all ages. In addition, in terms of region, the studies were conducted in four countries (United States, Denmark, Finland, and Sweden). The general characteristics of the included studies are presented in Table 1.

**Table 1:** Selected design characteristics of studies included in an analysis of the relation between asthma and IPD

Study	Study design	Country	Sample size	Population age	IPD definition
Talbot TR et al. (2005)	Case–control	USA	6985	2–49 years	Isolation of *S. pneumoniae* from a normally sterile site
James P. Watt et al. (2007)	Case–control	USA	471	> 18 years	Isolation of *S. pneumoniae* from a normally sterile site
Hjuler T et al. (2008)	Case–control	Denmark	17,028	0–17 years	Isolation of *S. pneumoniae* from a normally sterile site
Juhn YJ et al. (2008)	Case–control	USA	522	2–64 years	Isolation of *S. pneumoniae* from a normally sterile site or pneumococcal pneumonia requiring all three criteria
K. H. Yoo et al. (2009)	Case–control	USA	438	All ages	Isolation of *S. pneumoniae* from normally sterile body fluids
Klemets P et al. (2010)	Case–control	Finland	14,067	18–49 years	*S. pneumoniae* was isolated from blood and/or CSF
Pilishvili T et al. (2010)	Case–control	USA	3294	3–59 months	Isolation of Pneumococcus from a normally sterile site or a surveillance area resident
Inghammar M et al. (2013)	Case–control	Sweden	44,438	> 18 years	Isolation of *S. pneumoniae* from normally sterile body fluids

### Meta-analysis

**Fig. 2:**
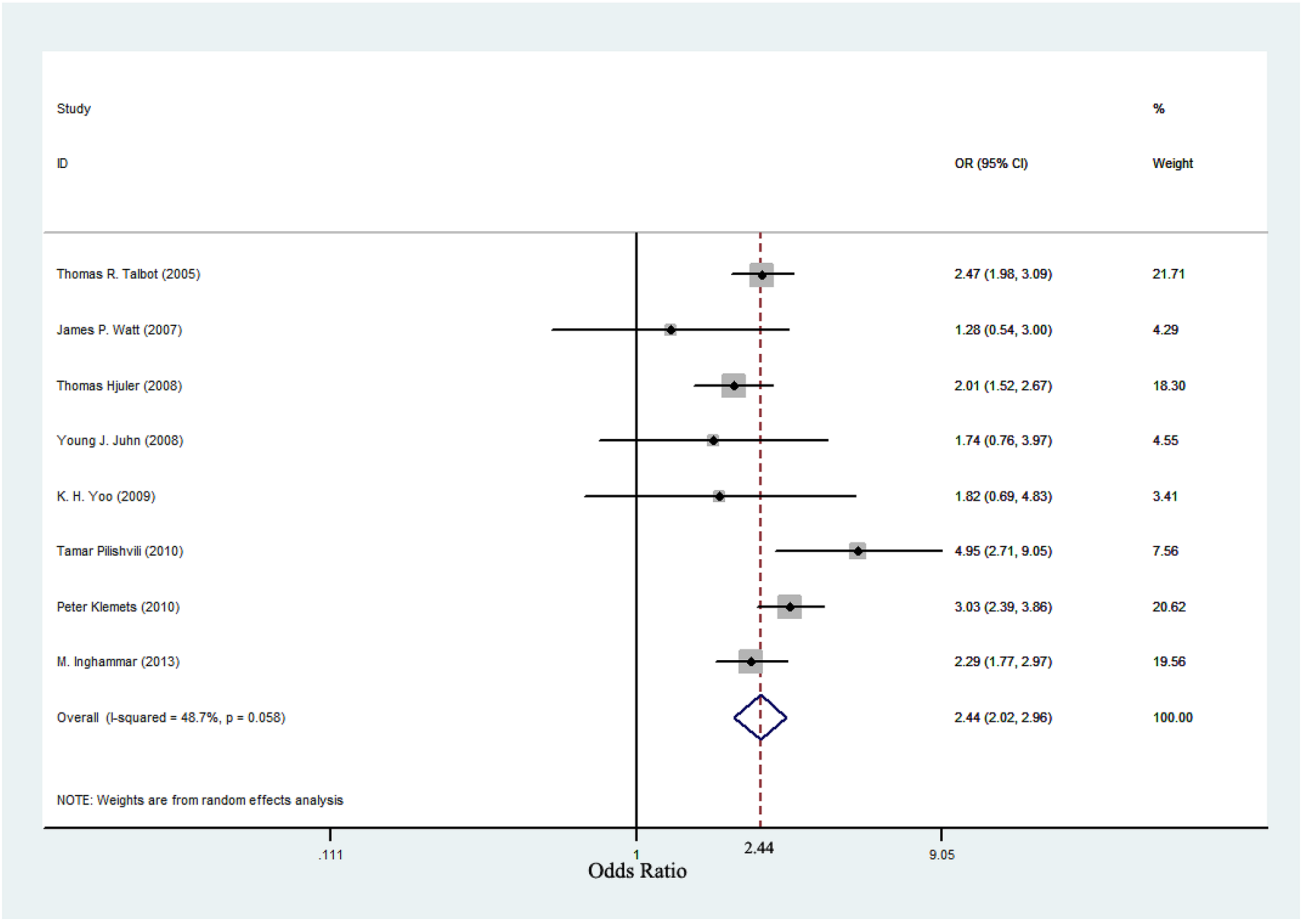
A meta-analysis of the prevalence of asthma in patients with IPD compared with controls. The odds ratios (OR) for asthma in subjects with IPD compared with subjects without IPD. Horizontal lines indicate 95% confidence intervals (CI). The pooled OR was analyzed by using a random-effects model

**Fig. 3:**
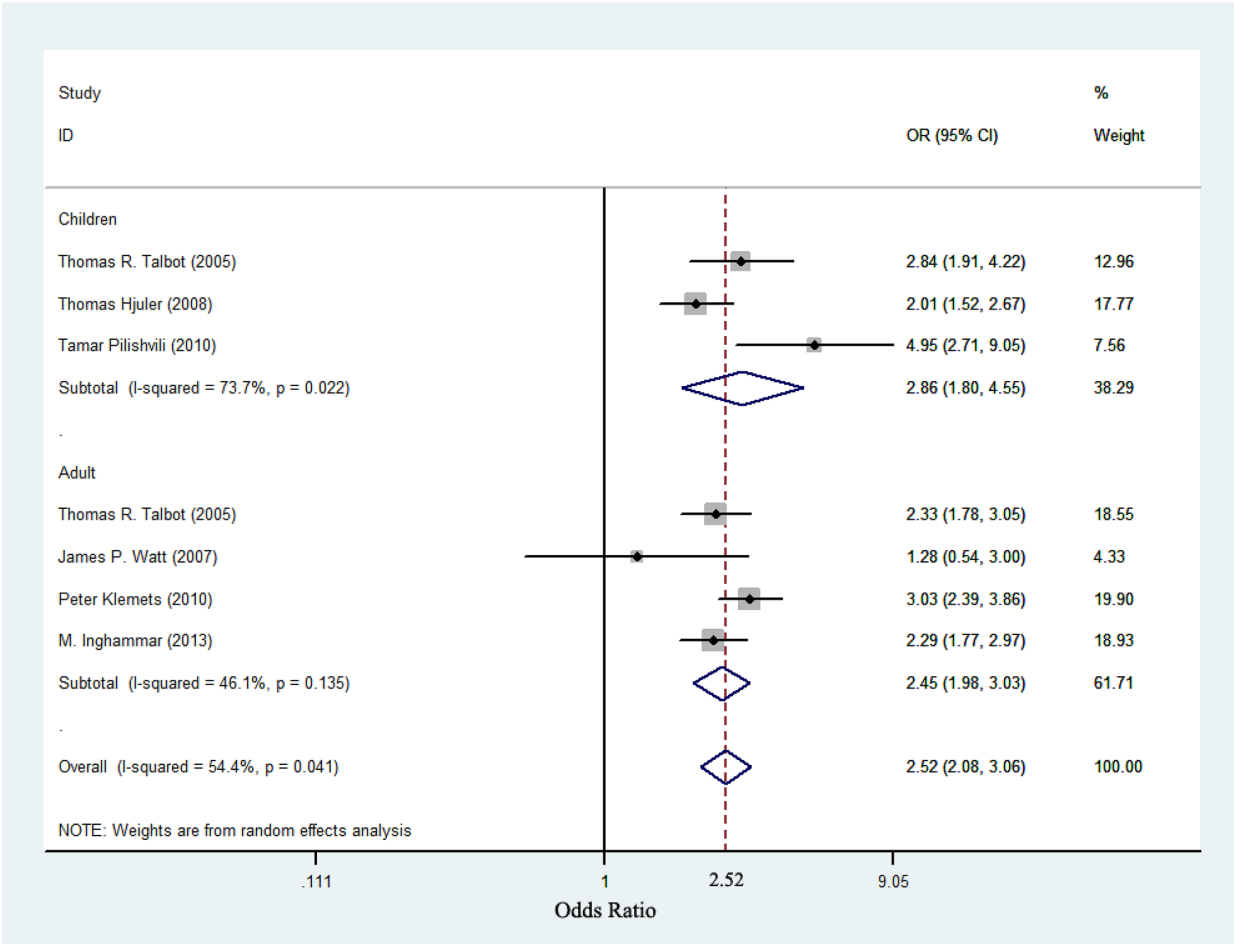
A meta-analysis of the prevalence of asthma in children and adults with IPD compared with controls. The odds ratios (OR) for asthma in subjects with IPD compared with subjects without IPD. Horizontal lines indicate 95% confidence intervals (CI). The pooled OR was analyzed by using a random-effects model

An assessment of heterogeneity of eight studies included for the analysis (I^2^ = 48.7%, *P* < 0.1), and the random-effects model was used to calculate the summary OR. Results are presented in Table 2 and Fig. 2. A significant common OR of 2.44 (95% CI 2.02–2.96, *P* < 0.001) was estimated, suggesting a significant positive association between asthma and IPD. When restricting the analysis of the association between asthma and IPD in adult, the OR was 2.45 (95% CI 1.98–3.03, *P* < 0.001) and the test result for heterogeneity was not significant (I^2^ = 46.7%, *P* > 0.1). However, when restricting the analysis of the association between asthma and IPD in children, the OR was 2.86 (95% CI 1.80–4.55, *P* < 0.001) and a significant heterogeneity was found (I^2^ = 73.7%, *P* < 0.1). The result is shown in Fig. 3.

**Table 2:** Results of included studies on the association between asthma and IPD

Study	Cases, n	Controls, n	Cases, No. with asthma	Controls, No. with asthma	OR (95% CI)
Talbot TR et al.	635	6350	114	516	2.47 (1.98, 3.09)
James P. Watt et al.	118	353	8	19	1.28 (0.54, 3.00)
Hjuler T et al.	1655	15,373	60	282	2.01 (1.52, 2.67)
Juhn YJ et al.	174	348	11	13	1.74 (0.76, 3.97)
K. H. Yoo et al.	146	292	8	9	1.82 (0.69, 4.83)
Klemets P et al.	1282	12,785	91	314	3.03 (2.39, 3.86)
Pilishvili T et al.	782	2512	27	18	4.95 (2.71, 9.05)
Inghammar M et al.	4085	40,353	71	309	2.29 (1.77, 2.97)

### Evaluation of publication bias

**Fig. 4:**
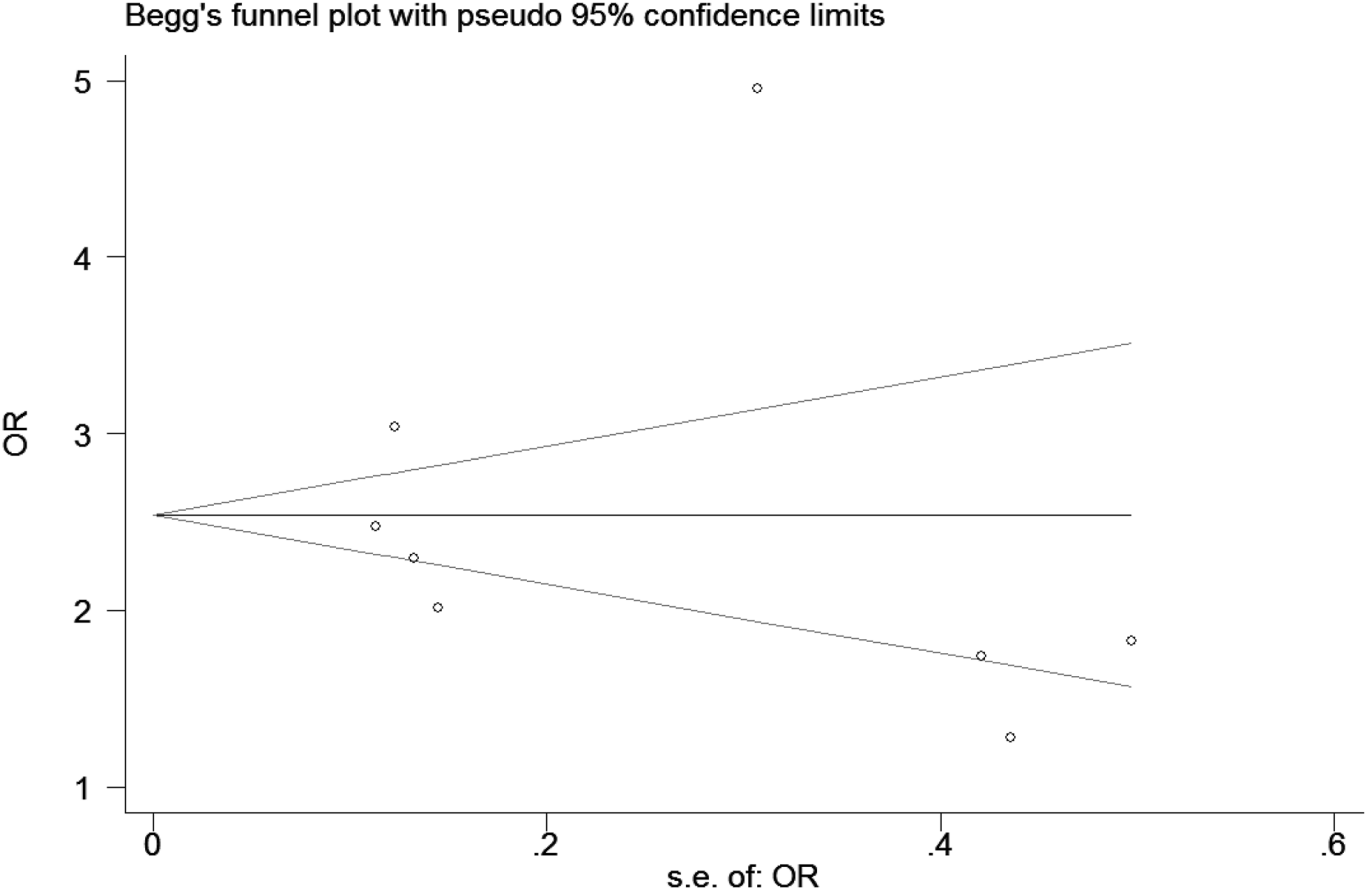
Publication bias in studies of the association between asthma and IPD assessed by the Begg funnel plot

Begg’s test was created for assessment of possible publication bias (Fig. 4). The *P* values for Egger’s tests were* P* = 0.942 (*P* > 0.1), indicating the absence of heterogeneity and implying that the results of the present meta-analysis were relatively stable and that the publication bias might exert little influence on the overall results.

## Discussion

We analyzed previous results on the potential association between asthma and IPD. Our results indicated that there was a significant association between asthma and IPD, patients with asthma had an increased risk for IPD susceptibility, and the risk was higher in children with asthma than in adults. To the best of the authors’ knowledge, this study is the first meta-analysis of the association between asthma and IPD.

Among the studies included, 8 indicated a significant association between asthma and IPD. Yoo et al. indicated asthma status was not associated with the risk of IPD in both adults and children adjusted for the high-risk conditions for IPD [24]. However, in only adults, asthma status was associated with the risk of IPD. In addition, A case–control study by Talbot et al. reported that a positive association persisted with a classification of high-risk asthma had 2.4 times the odds of IPD compared to controls while patients with a classification of low risk asthma had 1.7 times the odds of IPD [12]. In another study, Klemets et al. also indicated a higher risk for IPD susceptibility in high-risk asthmatics than low-risk asthmatics [16]. These two findings may emphasize the importance of taking into account the variability in risk of IPD among patients with asthma of different severity resulting from the type and intensity of treatments and the need for hospitalization.

In our study, we carried out subgroups analyses on different age groups, participants were divided into adults (≥ 18 years old) and children (0–17 years old). Four studies included patients of all ages, three studies included adult patients and two studies included children only. Finally, six studies provided detailed incidences of asthma in IPD patients at different ages. Random-effects models showed significant association between asthma and IPD for the children (OR 2.86, 95% CI 1.80–4.55) and adults (OR 2.45, 95% CI 1.98–3.03). The results suggest that asthma had a stronger association with IPD in children than in adults. This finding can be due to early life pneumococcal carriage leading to subsequent asthma. *S. pneumoniae* is commonly found in the nasopharynx, the source of primary spread [26] and usually the initial step towards infection. Carriage is most prevalent in children [27] and declines with increasing age [28], probably due to acquired immunity from exposure. Nasopharyngeal carriage is a dynamic process with changing prevalence, density and serotype of *S. pneumoniae*. A Prospective Study on Asthma in Childhood found that the infants were colonized with *S. pneumoniae* had an increased risk of a first wheezy episode, developing persistent wheeze or asthma during follow-up. Early nasopharyngeal carriage was also associated with increased blood eosinophil count, higher total serum IgE and airway reversibility. This study is strongly suggestive that early life nasopharyngeal carriage of *S. pneumoniae* is associated with subsequent asthma and the highly prevalence of pneumococcal carriage in children with asthma [29].

An association such as the one found between asthma and IPD may be due to a biological phenomenon and reflect a causal link or be due to a confounding factor not taken into account by statistical analyses [30]. We examined the biological relevance of predisposition to pneumococcal infections in asthmatic patients. Historically, studies of infections and asthma have centered on the impact of respiratory infections on asthma exacerbations, rather than infection susceptibility. More recent investigators have identified potential biologic mechanisms that may explain how asthma increases the risk of IPD. Unique pathologic alterations in the airway can lead to impaired clearance of pathogenic bacteria, implicating that the increased risk of invasive pneumococcal disease among persons with asthma has biologic plausibility. The respiratory epithelium and submucosal tissue of persons with asthma exhibit abnormal deposition of collagen and hyperplasia of goblet cells. The hyperplasia leads to increased production of mucin and alterations in secreted mucus, resulting in abnormalities in viscosity and in mucociliary clearance of the airway, increased production of sputum, and airway obstruction. The impaired clearance of airway debris can serve as a focus for localized infection that can develop into invasive bacterial infection. In the mouse sensitized to ovalbumin, inflammation and changes induced in the sinus mucosa by the inhalation of ovalbumin are associated to an increased frequency of pneumococcal infection after inoculation of the bacterium in the sinuses [31]. The hallmark of asthma is chronic airway inflammation mediated by innate and adaptive mechanisms [32]. The respiratory epithelium is the first line of defense against inhaled pathogenic agents and the bronchial epithelium of asthmatic patients is morphologically abnormal with areas of desquamation [33]. Frequent acute exacerbations of asthma, chronic airway inflammation and remodelling of the airways can be important features of asthma increasing the risk of microbial colonisation or infection [34]. In addition, persons with asthma have been shown to have increased rates of pneumococcal nasal colonization, as well as hypopharyngeal colonization as neonates before the development of asthma [20]. Impaired immune responses may account for increased rates of carriage and risk of invasive pneumococcal disease in asthma. Innate and adaptive immunity should be important determinants for protecting the host from microbial infection. Impaired innate immunity (impaired secretion of interferon-beta and -gamma by epithelial cells) in asthmatic patients has been recently demonstrated, which may make host susceptible to microbial infections, its relevance to the development of IPD [35, 36]. In adaptive immunity, asthma have been reported to be associated with a poor humoral immune response to pneumococcal polysaccharide vaccine [37, 38]. Numerous studies have demonstrated that type 2T helper cell (Th2) cytokines and reciprocally downregulated Th1 cells and functions have been suggested to be associated with susceptibility to and severity of microbial infections [39–42]. There are also genetic factors that affect patterns of immune response to infectious agents that are linked with asthma and asthma medications may be immunosuppressive [43]. Taken together, all of the above suggest an impaired immune response in people with asthma which may predispose to an increased risk of pneumococcal disease.

With the identification and now confirmation establishing asthma as a risk factor for invasive pneumococcal disease, known immune alerations that provide biological plausibility for increased susceptibility to infections among persons with asthma, we should reconsider whether persons with asthma should be candidates for pneumococcal vaccination. This issue is particularly important in view of the high and rapidly increasing prevalence of asthma in many parts of the world, and in the United States, affecting at least 8% of the population [44–47]. Guidelines for pneumococcal vaccination in asthma differ between adult and paediateic patient populations and between countries, and this can create confusion in clinical practice [48]. After widespread use of 7-valent pneumococcal conjugate vaccine (PCV7), IPD cases attributable to all pneumococcal serotypes decreased by 45%, and PCV7-serotypes decreases by 94% in all age groups in the US [49]. Currently the ACIP recommend vaccinating patients with chronic respiratory disease including asthma using the PPV [50]. The CDC recommends vaccination according to disease severity in adults, with PPV for mild asthma and PCV for severe disease [51]. However, it is remain unclear if this apparent blunted antibody response to vaccination results in reduced clinical benefit. Since there are limited data available for the role of pneumococcal vaccination in asthma, further studies are required to evaluate the burden of pneumococcal disease and the clinical effect of pneumococcal vaccination in patients with asthma.

There are several weaknesses in this meta-analysis. Firstly, our meta-analysis only focused on papers published in the English language and might miss some eligible studies that were unpublished in other languages, a publication bias may exist. Secondly, both asthma and IPD are complex diseases that may be misdiagnosed, and the diagnostic criteria of asthma or IPD is not exactly coherent in selected studies. Finally, despite using a precise literature searching strategy to identify eligible studies, it is possible that a few studies meeting the inclusion criteria were not included, resulting in any inevitable bias, though the Begg’s and Egger’s tests failed to show any significant publication bias. These factors could affect the results, which would show no statistically significant association between asthma and IPD.

## Conclusion

In conclusion, the current meta-analysis indicated that patients with asthma had an increased risk of IPD susceptibility, the risk was higher in children with IPD than in adult patients. Further investigations on the immunologic and molecular mechanisms that can explain the association are needed to understand the relation between asthma and IPD. These results highlights the need for timely pneumococcal vaccination in persons with asthma.

## Data Availability

Not applicable.
